# Correction to: The NYCKidSeq project: study protocol for a randomized controlled trial incorporating genomics into the clinical care of diverse New York City children

**DOI:** 10.1186/s13063-021-05057-3

**Published:** 2021-02-16

**Authors:** Jacqueline A. Odgis, Katie M. Gallagher, Sabrina A. Suckiel, Katherine E. Donohue, Michelle A. Ramos, Nicole R. Kelly, Gabrielle Bertier, Christina Blackburn, Kaitlyn Brown, Lena Fielding, Jessenia Lopez, Karla Lopez Aguiniga, Estefany Maria, Jessica E. Rodriguez, Monisha Sebastin, Nehama Teitelman, Dana Watnick, Nicole M. Yelton, Avinash Abhyankar, Noura S. Abul-Husn, Aaron Baum, Laurie J. Bauman, Jules C. Beal, Toby Bloom, Charlotte Cunningham-Rundles, George A. Diaz, Siobhan Dolan, Bart S. Ferket, Vaidehi Jobanputra, Patricia Kovatch, Thomas V. McDonald, Patricia E. McGoldrick, Rosamond Rhodes, Michael L. Rinke, Mimsie Robinson, Arye Rubinstein, Lisa H. Shulman, Christian Stolte, Steven M. Wolf, Elissa Yozawitz, Randi E. Zinberg, John M. Greally, Bruce D. Gelb, Carol R. Horowitz, Melissa P. Wasserstein, Eimear E. Kenny

**Affiliations:** 1 The Institute for Genomic Health, Icahn School of Medicine at Mount Sinai, New York, NY USA; 2 Department of Pediatrics, Division of Pediatric Genetic, Medicine, Children’s Hospital at Montefiore/Montefiore Medical Center/Albert, Einstein College of Medicine, Bronx, NY USA; 3 Department of Population, Health Science and Policy, Icahn School of Medicine at Mount Sinai, New York, NY USA; 4 Institute for Health Equity Research, Icahn School of Medicine at Mount Sinai, New York, NY USA; 5 Molecular Diagnostics, New York, Genome Center, New York, NY USA; 6 Department of Pediatrics, Division of, Pediatric Academic Medicine, Children’s Hospital at Montefiore/Montefiore, Medical Center/Albert Einstein College of Medicine, Bronx, NY USA; 7 Department of Genetics and Genomic Sciences, Icahn School of Medicine at Mount Sinai, New York, NY USA; 8 Department of Medicine, Icahn School of Medicine at Mount Sinai, New York, NY USA; 9 Department of Health System, Design and Global Health, Icahn School of Medicine at Mount Sinai, New York, NY USA; 10 Department of Pediatrics, Division of Child Neurology, Weill Cornell Medical, New York, NY USA; 11 Department of Pediatrics, Icahn School, of Medicine at Mount Sinai, New York, NY USA; 12 Department of Obstetrics and Gynecology and Women’s Health (Reproductive and Medical Genetics), Albert Einstein College of Medicine, Bronx, NY USA; 13 Department of Pathology and Cell Biology, Columbia University Medical Center, New York, NY USA; 14 Scientific Computing and Data Science, Icahn School of Medicine at Mount Sinai, New York, NY USA; 15 Department of Medicine (Cardiology), Montefiore/Montefiore Medical Center/Albert Einstein College of Medicine, Bronx, NY USA; 16 Department of Pediatrics, Division of Child Neurology, New York Medical College, Valhalla, NY USA; 17 Pediatric Neurology|Boston Children’s Health Physicians/Maria Fareri Children’s Hospital, Hawthorne, NY USA; 18 Department of Medical Education, Icahn School of Medicine at Mount Sinai, New York, NY USA; 19 Bethel Gospel Assembly, New York, NY USA; 20 Department of Microbiology and Immunology, Albert Einstein College of Medicine, Bronx, NY USA; 21 Isabelle Rapin Division of Child Neurology of the Saul R Korey Department of Neurology at Montefiore Medical Center/Albert Einstein College of Medicine, Bronx, NY USA; 22 Department of Obstetrics, Gynecology and Reproductive Science, Icahn School of Medicine at Mount Sinai, New York, NY USA; 23 Mindich Child Health and Development Institute, Icahn School of Medicine at Mount Sinai, New York, NY USA

**Correction to: Trials 22, 56 (2021)**

**https://doi.org/10.1186/s13063-020-04953-4**

Following publication of the original article [1], we were notified that the originally published Table 2 was incorrect. References 1–33 were not impacted by the table update and remain unchanged, but are updated from reference 34 onward.

- Originally published Table 2

**Table 2:** NYCKidSeq participant outcomes by survey timepoint

Variable	Source	BL^**1**^	ROR1^**2**^	ROR2^**3**^
**Primary outcome**				
*Perceived understanding of genomic testing results*	NYCKidSeq developed measure (novel)	**–**	**X**	**X**
**Secondary outcomes**				
*Objective understanding of genomic testing results*	NYCKidSeq developed measure (novel)	**–**	**X**	**X**
*Medical actions and non-medical/patient-initiated actions attributable to genomic testing*	CSER developed measures (novel): Attributable to Genomic Testing (RMA) and Patient-Initiated Actions Attributable to Genomic Testing (PIA)	**–**	**–**	**X**
**Attitudes**				
*Satisfaction with the mode of delivery*	CSER developed measure (novel) adapted from Patient Assessment of cancer Communication Experiences (PACE) [34, 35]	**–**	**X**	**–**
*Satisfaction with results*	Satisfaction with information about medicine (SIMS) [36]	**–**	**X**	**–**
*Attitudes toward genetic testing*	Adapted from Genetic testing to Understand and Address Renal Disease Disparities (GUARDD) study [37, 38]	**X**	**X**	**X**
*Empowerment*	Adapted from GUARDD study [37]	**X**	**X**	**X**
*Decisional conflict*	Decisional Conflict Scale (Low Literacy) [39]	**X**	**X**	**X**
**Perceived utility**				
*Impact of genomic testing on health status*	Functional status II-R (child) [40]	**X**	**–**	**X**
*Impact of genomic testing on quality of life*	Child Health Utility Instrument (CHU9D; parent as proxy) [41]; SF-12 health survey (for parent) [42]	**X**	**–**	**X**
*Clinical utility*	Patient-reported utility (PrU) [43]	**–**	**X**	**X**
**Psychological impact**				
*Feelings about genomic testing results*	Feelings About Genomic Testing Results (FACToR) [44]	**–**	**X**	**X**
*Uncertainty*	Perceptions of Uncertainties in Genomic Sequencing (PUGS) [45]; FACToR subscale [44]	**–**	**X**	**X**
*Depression*	8-item Patient Health Questionnaire depression scale (PHQ-8) [46]	**X**	**X**	**X**
*Anxiety*	Generalized Anxiety Disorder Screener (GAD-2) [47, 48]	**X**	**X**	**X**
*Perceived stress*	Perceived Stress Scale 4-item (PSS-4) [49]	**X**	**X**	**X**
*Self-efficacy*	Decision Self-Efficacy Scale [50]	**X**	**–**	**–**
*Patient activation*	Short Form Patient Activation Measure (PAM) [51]	**X**	**–**	**–**
*Decisional regret*	Decision Regret Scale [52]	**–**	**X**	**X**
**Behavioral impact**				
*Information seeking*	CSER developed measure (novel); Adapted from Psychological Adaptation to Genetic Information Scale [53]	**–**	**X**	**X**
*Family communication*	CSER developed measure (novel)	**–**	**–**	**X**
**Social impact**				
*Support*	Low-Literacy Decisional Conflict Scale (Q6 and Q8) [54]	**X**	**X**	**X**
*Access to care*	CSER developed measure (novel)	**X**	**X**	**X**
*Life chaos*	Chaos Scale [55]	**X**	**–**	**–**
*Family and community*	Medical Outcomes Study Social Support Survey (mMOS-SS) [56]	**X**	**–**	**–**
*Quality of life ascertainment (for child)*	PedsQL Parent Proxy Generic Core [57]; EuroQol-Visual Analog Scale (VAS) [58]	**X**	**–**	**X**
**Economic impact**				
*Cost/value*	CSER developed measure (novel)	**–**	**X**	**X**
*Healthcare utilization*	Self-reported Utilization of Health Care Services [59]	**–**	**X**	**X**
**Sociodemographic factors**				
*Literacy; numeracy*	BRIEF Health Literacy Survey [60]; Subjective Numeracy Scale (SNS-3) [61]	**X**	**–**	**–**
*History of receiving genetic testing*	Adapted from the GUARDD study [37]	**X**	**–**	**–**
*Trust in healthcare system*	CSER developed measure (novel) adapted from Health Care System Distrust Scale [62]	**X**	**–**	**–**
*Health beliefs*	Brief Illness Perception Questionnaire (IPQ) [63]	**X**	**–**	**–**
*Child and parent: sex, age, race/ethnicity, country of origin, language, insurance status, residential history, zip code*	CSER developed measure (novel); Adapted from HCHS/SOL Personal Information Questionnaire [64]	**X**	**–**	**–**
*Parent only: education level, employment, income, household, marital status*	CSER developed measure (novel); Adapted from HCHS/SOL Personal Information Questionnaire [64]	**X**	**–**	**–**
*Grandparents of child: residential history*	Adapted from HCHS/SOL Personal Information Questionnaire [64]	**X**	**–**	

^1^BL = baseline survey

^2^ROR1 = return of results, visit 1 survey

^3^ROR2 = return of results, visit 2 survey

- Corrected Table 2

**Table 2:** NYCKidSeq participant outcomes by survey timepoint

VARIABLE	SOURCE^**a**^	BL^**b**^	ROR1^**c**^	ROR2^**d**^
**Understanding**				
*Perceived understanding of genomic testing results*	NYCKidSeq (novel); CSER (novel); CSER measure adapted from Psychological Adaptation to Genetic Information Scale (PAGIS) [35]	**–**	**X**	**X**
*Objective understanding of genomic testing results*	NYCKidSeq (novel)	**–**	**X**	**X**
*Understanding of medical follow up & actionability*	Adapted from CSER (novel): Recommended Medical Actions and Follow Through on Recommendations Attributable to Genomic Testing (MRA)	**–**	**X**	**–**
**Attitudes**				
*Expectations of genetic testing*	Adapted from Patient Reported Utility (PrU) [36]; NYCKidSeq (novel)	**X**	**–**	**–**
*Satisfaction with results and communication mode*	CSER (novel)	**–**	**X**	**–**
*Patient assessment of communication*	CSER measure adapted from Patient Assessment of cancer Communication Experiences (PACE) [37, 38]	**–**	**X**	**–**
*Evaluation of communication tool (GUÍA)*	NYCKidSeq (novel) adapted from Lobb et al. 2006 [39) and Sanderson et al. 2016 [40]	**–**	**X**	**–**
*Satisfaction with interpretation and perceived cultural concordance (Spanish speakers only)*	CSER (novel)	**–**	**X**	**–**
*Evaluation of provided patient resources*	NYCKidSeq (novel)	**–**	**–**	**X**
**Perceived Utility**				
*Patient reported utility*	CSER measure adapted from Patient Reported Utility (PrU) [36]	**–**	**X**	**X**
**Psychological Impact**				
*Feelings about genomic testing results*	CSER measure adapted from Feelings About Genomic Testing Results (FACToR) [41]	**–**	**X**	**X**
*Uncertainty*	CSER measure adapted from Perceptions of Uncertainties in Genomic Sequencing (PUGS) [42] and FACToR subscale [41]	**–**	**X**	**X**
*Decisional regret (for positive secondary findings only)*	Adapted from Decision Regret Scale [43]	**–**	**X**	**X**
**Behavioral Impact**				
*Information seeking*	CSER (novel)	**–**	**X**	**X**
***Adherence to medical follow up recommendations; Patient-Initiated actions attributable to genomic testing***	CSER (novel): Recommended Medical Actions and Follow Through on Recommendations Attributable to Genomic Testing (MRA); Patient-Initiated Actions Attributable to Genomic Testing (PIA)	**–**	**–**	**X**
*Family communication*	CSER (novel)	**–**	**–**	**X**
**Social Impact**				
*Access to care*	CSER measure adapted from Medicare Expenditure Panel Survey, Household Component (MEPS-HC) [44]	**X**	**–**	**–**
*Quality of life ascertainment (for child)*	Pediatric Quality of Life Inventory (PedsQL) Parent Proxy Generic Core [45]; Adapted from EuroQol-Visual Analog Scale (VAS) [46]	**X**	**–**	**X**
**Economic Impact**				
*Cost utility*	Adapted from Hebert et al. 2008 [47] and Valuation of Informal Care Questionnaire (iVICQ) [48]	**X**	**–**	**X**
**Sociodemographic Factors**				
*Health literacy; Subjective numeracy*	CSER measure adapted from BRIEF Health Literacy Survey [49]; CSER measure adapted from Subjective Numeracy Scale (SNS-3) [50]	**X**	**–**	**–**
*History of receiving genetic testing*	NYCKidSeq (novel) adapted from Genetic testing to Understand and Address Renal Disease Disparities (GUARDD) study [51]	**X**	**–**	**–**
*Trust in health care system*	CSER measure adapted from Health Care System Distrust Scale [52]	**X**	**–**	**–**
*Insurance status of child*	CSER measure adapted from National Health and Nutrition Examination Survey (NHANES) [53]	**X**	**–**	**X**
*Child only: sex, grandparent(s) country of origin*	CSER measure adapted from GenIUSS [54], CSER (novel)	**X**	**–**	**–**
*Child and Parent: age, race/ethnicity, country of origin, zip code*	Date of birth, CSER measure adapted from US Census [55, 56], CSER (novel), Zip code	**X**	**–**	**–**
*Parent only: education level, language, income, household, marital status*	Education and language: CSER (novel) Income and household: CSER measure adapted from NHANES [53] Marital status: NYCKidSeq (novel)	**X**	**–**	**–**

^a^Note: NYCKidSeq measures were developed specifically for the RCT. CSER measures were developed by a collaborative group of CSER investigators, as outlined in Goddard et al., 2020 [56]

^b^*BL* Baseline survey

^c^*ROR1* Return of results, visit 1 survey

^d^*ROR2* Return of results, visit 2 survey

**REFERENCES:**

34. Read CY, Perry DJ, Duffy ME. Design and psychometric evaluation of the Psychological Adaptation to Genetic Information Scale. J Nurs Scholarsh. 2005;37(3):203–8.

35. Kohler JN, Turbitt E, Lewis KL, Wilfond BS, Jamal L, Peay HL, et al. Defining personal utility in genomics: A Delphi study. Clin Genet. 2017 Sep;92(3):290–7.

36. Mazor KM, Street RL Jr, Sue VM, Williams AE, Rabin BA, Arora NK. Assessing patients’ experiences with communication across the cancer care continuum. Patient Educ Couns. 2016 Aug;99(8):1343–8.

37. Street RL Jr, Mazor KM, Arora NK. Assessing Patient-Centered Communication in Cancer Care: Measures for Surveillance of Communication Outcomes. J Oncol Pract. 2016 Dec;12(12):1198–202.

38. Lobb EA, Butow PN, Moore A, Barratt A, Tucker K, Gaff C, et al. Development of a communication aid to facilitate risk communication in consultations with unaffected women from high risk breast cancer families: a pilot study. J Genet Couns. 2006;15:393–405.

39. Sanderson SC, Suckiel SA, Zweig M, Bottinger EP, Jabs EW, Richardson LD. Development and preliminary evaluation of an online educational video about whole-genome sequencing for research participants, patients, and the general public. Genet Med. 2016;18:501–12.

40. Li M, Bennette CS, Amendola LM, Ragan Hart M, Heagerty P, Comstock B, et al. The Feelings About genomiC Testing Results (FACToR) Questionnaire: Development and Preliminary Validation. J Genet Couns. 2019 Apr;28(2):477–90.

41. Biesecker BB, Woolford SW, Klein WMP, Brothers KB, Umstead KL, Lewis KL, et al. PUGS: A novel scale to assess perceptions of uncertainties in genome sequencing. Clin Genet. 2017 Aug;92(2):172–9.

42. Brehaut JC, O’Connor AM, Wood TJ, Hack TF, Siminoff L, Gordon E, et al. Validation of a decision regret scale. Med Decis Making. 2003 Jul;23(4):281–92.

43. Agency for Healthcare Research and Quality (AHRQ). Medicare Expenditure Panel Survey (MEPS)-Household Component (HC), Access to Care Section (P18R5/P19R3/P20R1), Variable: Recommended Family Testing & Monitoring. US Department of Health and Human Services.

44. Varni JW, Seid M, Kurtin PS. PedsQL^TM^ 4.0: Reliability and Validity of the Pediatric Quality of Life Inventory^TM^ Version 4.0 Generic Core Scales in Healthy and Patient Populations. Med Care. 2001 Aug;39(8):800.

45. Wille N, Badia X, Bonsel G, Burström K, Cavrini G, Devlin N, et al. Development of the EQ-5D-Y: a child-friendly version of the EQ-5D [Internet]. Quality of Life Research. 2010. p. 875–86. Available from: 10.1007/s11136-010-9648-y

46. Hebert PL, Sisk JE, Wang JJ, Tuzzio L, Casabianca JM, Chassin MR, et al. Cost-effectiveness of nurse-led disease management for heart failure in an ethnically diverse urban community. Ann Intern Med. 2008;149:540–8.

47. Hoefman RJ, Van Exel NJA, Brouwer WBF. iMTA Valuation of Informal Care Questionnaire (iVICQ). Version 1.0 (December 2011). Rotterdam: iBMG / iMTA, 2011. [retrieved from www.bmg.eur.nl/english/imta/publications/manuals_questionnaires/ on 01/12/2021]

48. Haun, J., Noland Dodd, V. J., Graham-Pole, J., Rienzo, B., & Donaldson, P. (2009). Testing a Health Literacy Screening Tool: Implications for Utilization of a BRIEF Health Literacy Indicator. Federal Practitioner, 26(12), 24-31.

49. McNaughton CD, Cavanaugh KL, Kripalani S, Rothman RL, Wallston KA. Validation of a Short, 3-Item Version of the Subjective Numeracy Scale [Internet]. Vol. 35, Medical Decision Making. 2015. p. 932–6. Available from: 10.1177/0272989x15581800

50. Horowitz CR, Abul-Husn NS, Ellis S, Ramos MA, Negron R, Suprun M, et al. Determining the effects and challenges of incorporating genetic testing into primary care management of hypertensive patients with African ancestry. Contemp Clin Trials. 2016 Mar;47:101–8.

51. Shea JA, Micco E, Dean LT, McMurphy S, Sanford Schwartz J, Armstrong K. Development of a Revised Health Care System Distrust Scale [Internet]. Vol. 23, Journal of General Internal Medicine. 2008. p. 727–32. Available from: 10.1007/s11606-008-0575-3

52. United States Department of Health and Human Services. Centers for Disease Control and Prevention. National Center for Health Statistics. National Health and Nutrition Examination Survey (NHANES), 1999-2000 [Internet]. ICPSR Data Holdings. 2009. Available from: 10.3886/icpsr25501.v3

53. The GenIUSS Group. Best Practices for Asking Questions to Identify Transgender and Other Gender Minority Respondents on Population- Based Surveys. Los Angeles, CA: The Williams Institute, 2014.

54. Jones NA. Update on the U.S. Census Bureau’s Race and Ethic Research for the 2020 Census. Survey News 2015;3(5). (https://www.census.gov/content/ dam/Census/newsroom/press-kits/2014/article_race_ethnic_research_2020census_jones.pdf)

55. Matthews K, Phelan J, Jones NA, Konya S, Marks R, Pratt BM, et al. 2015 national content test race and ethnicity analysis report: A new design for the 21st century. US Census Bureau Washington, DC; 2017.

56. Goddard KAB, Angelo FAN, Ackerman SL, Berg JS, Biesecker BB, Danila MI, et al. Lessons learned about harmonizing survey measures for the CSER consortium. Journal of Clinical and Translational Science. Cambridge University Press; 2020;4:537–46.

The original article has been corrected.
